# Host–Virus Interactions in Feline Kidney Cells Infected with a Chinese Epidemic Strain of Feline Panleukopenia Virus Analysed Using RNA-Seq

**DOI:** 10.3390/vetsci12080748

**Published:** 2025-08-12

**Authors:** Erkai Feng, Shun Wu, Shipeng Cheng, Yuening Cheng

**Affiliations:** Institute of Special Animal and Plant Science, Chinese Academy of Agriculture Science, Changchun 130112, China; fengerkai@caas.cn (E.F.); 13271660260@163.com (S.W.)

**Keywords:** FPLV, RNA-seq, virus–host interaction, innate immune, signaling pathway

## Abstract

Feline panleukopenia virus (FPLV) is a significant causative agent of disease in both domestic cats and wild carnivores that poses a considerable threat to their health. Despite its clinical importance, the mechanisms underlying FPLV–host interactions remain poorly understood. In this study, we conducted a systematic analysis of transcriptomic changes in feline kidney cells (F81) infected with a Chinese FPLV strain using RNA-seq. The down-regulated differentially expressed genes (DEGs) were majorly enriched in the regulation of the cell cycle, cell growth, or cell senescence, while the up-regulated DEGs were found to be significantly associated with cellular pathways involved in cell cycle regulation, extrinsic apoptotic signaling, and key host immune responses, including Toll-like receptor, JAK-STAT, IL-17, and TNF signaling pathways. By validating the RNA-seq data with RT-qPCR (real-time quantitative PCR) results, we identified potentially important immune-associated genes involved in the host immune response to feline panleukopenia virus, including IGSF6, IFI44L, IFI6, IFITM10, IL1R1, and JAK3. Overall, our results provide valuable insights into the mechanisms underlying feline panleukopenia virus and its interactions with its host, laying the foundation for future research on this significant virus and its impact on feline health.

## Simple Summary

Feline panleukopenia virus (FPLV) is a significant causative agent of disease in both domestic cats and wild carnivores. However, despite circulating for more than 100 years, there have been no reports on FPLV–host interactions. Therefore, we systematically analysed transcriptomic changes in feline kidney cells (F81) infected with a Chinese FPLV epidemic strain using RNA-seq. Overall, 3116 differentially expressed genes (DEGs) were identified in this study, with 1733 genes up-regulated and 1383 genes down-regulated. The down-regulated DEGs were majorly enriched in the cell cycle, cell growth, or cell senescence, while the up-regulated DEGs were found to be involved in regulating the extrinsic apoptotic signaling and some key mechanisms in host defence against FPLV infections, such as signaling pathways involving Toll-like receptors, JAK-STAT, IL-17, and TNF. Potentially important immune-associated genes, such as IGSF6, IFI44L, IFI6, IFITM10, IL1R1, and JAK3, were also identified. These may play a vital role in the host immune response to feline panleukopenia virus.

## 1. Introduction

Feline panleukopenia virus (FPLV) is a crucial pathogen that poses a significant threat to the well-being of cats and significantly contributes to the rising expenses of pet healthcare, thereby demanding immediate attention and effective management measures [1]. The clinical manifestations of FPLV infection are alarming, including a sudden onset of fever, vomiting, diarrhoea, dehydration, panleukopenia, and, eventually, death [2]. Despite being initially identified in the 1920s [3] and isolated in 1965 [4], understanding of its pathogenicity and the molecular mechanisms underlying host antiviral response remains inadequate. As recently as 2019, an FPLV strain with a VP2 A91S substitution caused a widespread pandemic in China, leading to a significant increase in morbidity and mortality rates. This event garnered substantial attention from veterinary researchers, who deemed it an essential and vital health concern due to its potential impact on the feline population [5,6].

As obligate intracellular parasites, viruses are closely intertwined with their host cells [7]. During this period, numerous biological changes occur, including the host’s innate and adaptive immune responses to the virus as well as alterations in the metabolic and energetic state of the infected cell [8]. A crucial aspect of understanding phenotypic variations is the accurate identification of differentially expressed genes (DEGs) under specific conditions, such as between naïve and virus-infected cells.

High-throughput transcriptome sequencing (RNA-seq) is the preferred method for elucidating gene expression dynamics. Fan XX and his colleagues demonstrated that CPV-2a infection triggers a substantial number of differentially expressed genes (DEGs) closely linked to various signaling pathways involved in virus–host interactions [9]. Furthermore, *S. Vannamahaxay* and his team identified 11 genes (C1QBP, CD40, HYAL2, IFNB1, IFNG, IL12B, IL6, IRF3, LSM14A, MAVS, and NLRC5) associated with the defence response to CPV-2c [10]. These studies underscore the significance of understanding the relationship between viruses and their host cells. However, to the best of our knowledge, there are limited reports on host–virus interactions for feline parvoviruses, with a report on early transcriptional changes in feline herpesvirus-1-infected CRFK cells [11].

The primary aim of this study is to conduct a comprehensive analysis of the intricate relationship between FPLV and host cells. This is achieved by comparing the transcriptome profiles of FPLV-infected F81 cells with those of uninfected cells using high-throughput RNA sequencing (RNA-seq) technology.

## 2. Materials and Methods

### 2.1. Virus and Cells

*Felis catus* kidney cells (F81 cells) were purchased from the Cell Resource Center of the Shanghai Institute of Biological Sciences at the Chinese Academy of Sciences and maintained in minimum essential medium (MEM) with 10% foetal bovine serum (FBS, Gibco, Waltham, MA, USA) in an incubator in 5% CO_2_ at 37 °C.

FPLV-CC19-02, feline panleukopenia virus carrying a VP2 A91S substitution (GenBank number: OR921195.1), was previously isolated from a cat suffering from severe diarrhoea in Changchun city, Jilin Province, by serially passaging the treated faecal sample on F81 cells, which was further characterized by typical cytopathic effect (CPE), PCR identification, and direct or indirect immunofluorescence analyses.

### 2.2. Sample Preparation, Library Construction, and RNA-Sequencing

A 10 mL suspension of F81 cells (5 × 10^5^ cells/mL) was initially seeded into T75 cell culture flasks (Costar, Kenilworth, NJ, USA) and maintained in an incubator in 5% CO_2_ at 37 °C for 24 h. Then, F81 cells were infected with FPLV at a multiplicity of infection (MOI) of 1. Finally, infected cells were harvested at various time points, 0, 6, 12, 24, and 48 h post-inoculation (hpi), using cell scrapers and stored in TRIzol Reagent (Ambion, Thermo Fisher, Waltham, MA, USA). F81 cells harvested at zero h served as a negative control (mock). Three biological replicates were analysed for each time point to ensure accuracy.

The growth kinetics of FPLV in F81 cells were analysed using real-time quantitative PCR with a pair of primers that target the VP2 sequence of FPLV. The cytopathic effect (CPE) of FPLV-infected cells was also recorded.

The total RNA was extracted from uninfected (mock) and infected F81 cells using a combination of RNAzol^®^ RT reagent (Sigma-Aldrich, St. Louis, MO, USA) and the Direct-Zol^TM^ RNA Miniprep kit (Zymo Research, Irvine, CA, USA). A total of 5 μL DNase I (6 U/μL) and 75 μL DNA digestion buffer was added and incubated at room temperature for 15 min to eliminate genome DNA contamination. NanoDrop Qubit 2.0 (Thermo Fisher Scientific, Waltham, MA, USA), RNA agarose electrophoresis, and Agilent bioanalyser 2100 (Agilent Technologies, Santa Clara, CA, USA) were used to assess the quality and integrity of the extracted RNA.

Sequencing libraries were prepared using the NEBNext Ultra RNA Library Prep Kit for Illumina^®^ (NEB, Ipswich, MA, USA), following the manufacturer’s guidelines. Briefly, the dual goals of removing rRNA from total RNA and enriching mRNA were fulfilled using an isolation approach based on NEBNEXT Poly (A) mRNA magnetic beads (Oligo dT). Then, the purified mRNA was randomly fragmented into small pieces by treatment with divalent cations for 15 min at 94 °C. The fragmented mRNA was then subjected to reverse transcription using a NEB random primer in a cDNA synthesis reaction. The double-stranded cDNA was purified and then subjected to double-end repair, with an “A” base added onto the 3′ end. Illumina paired-end adapters were then ligated to the ends. The adapter-ligated cDNA was then enriched by PCR amplification and purified using AMPure XP beads (BECKMAN Coulter Life Science, Shanghai, China).

Libraries with concentrations exceeding 2 nM are considered suitable. Following cluster generation, the library preparations were sequenced on an Illumina Hiseq^®^ platform, producing 150 bp paired-end reads.

### 2.3. RNA-Sequencing Data Quality Control and Analysis

Clean reads were extracted from the raw data in FASTQ format by removing reads containing adapters or poly-N sequences as well as those of low quality. The Phred quality scores (Q score) of Q20 and Q30 were calculated for the cleaned data. Subsequently, reads found to be of high quality were aligned to the *Felis catus* reference genome [12] (Table 1) using HISAT2 version 2.2.1.

### 2.4. Gene Expression Analysis

Gene expression levels were quantified by tallying reads mapped to each gene using HTSeq (version 0.6.1) [13]. Values of fragments per kilobase per million fragments (FPKM) were then calculated for each gene based on its length and the corresponding mapped read count [14].

Inter-sample correlation of gene expression levels is a critical metric for assessing experimental reliability and the rationality of sample selection. The Pearson correlation coefficient was used to quantify inter-sample correlations in gene expression levels.

Principal component analysis (PCA) was conducted using the DESeq2 package in R, based on gene expression levels across samples.

### 2.5. Differentially Expressed Genes (DEGs) Analysis

Differential expression analysis was performed between the infected and control groups, utilising the DESeq2 R package (version 1.20.0). Genes were considered differentially expressed if they had a |log2Fold Change| value greater than 1 [15,16] and a *p*-value (FDR-adjusted) of less than 0.05.

#### 2.5.1. Clustering Analysis

The R language Pheatmap software package was used to perform bidirectional clustering analysis of differentially expressed genes across all comparison groups. We performed clustering based on the expression level of a specific gene across various samples and the expression patterns of diverse genes within the same sample. To calculate the distance, we utilised the Euclidean method.

#### 2.5.2. Gene Ontology (GO) Enrichment Analysis of DEGs

To further explore the functional roles of the differentially expressed genes (DEGs), gene ontology (GO) enrichment analysis was performed using the ClusterProfiler R package. This analysis was performed while accounting for gene length bias, and GO terms were considered significantly enriched if they had a corrected *p*-value of below 0.05.

#### 2.5.3. Kyoto Encyclopedia of Genes and Genomes (KEGG) Enrichment Analysis of DEGs

The statistical enrichment of differentially expressed genes (DEGs) in KEGG pathways was evaluated using the ClusterProfiler R package (version 4.16). The KEGG database (http://www.genome.jp/kegg/, accessed on 12 March 2023) was used to investigate the biological functions of these DEGs.

### 2.6. Real-Time Quantitative PCR (RT-qPCR) Validation of the RNA-Seq

The status of six selected differentially expressed genes (IGSF6, IFI44L, IFI5, IFITM10, IL1R1, and JAK3) was confirmed using quantitative real-time PCR (qPCR). The house-keeping gene GAPDH (glyceraldehyde-3-phosphate dehydrogenase) was chosen as an internal control for normalisation in gene expression studies. The primers were designed by using NBCI’s primer designing tool (Primer-BLAST, https://blast.ncbi.nlm.nih.gov/Blast.cgi, accessed on 15 September 2024) and are shown in Table 2. Total RNA was extracted and purified from each sample as stated earlier in Section 2.2.

First-strand cDNA was synthesised using MonScript™ RTIII All-in-One Mix with DNase (Monad, Suzhou, China). RT-qPCR assays were performed on an ABI 7500 quantitative PCR instrument (Applied Biosystems Inc., Waltham, MA, USA) with the following conditions: the reaction volume was 10 µL; 2x SYBR Green PCR Master Mix, 5 µL; ROX, 0.05 µL; each primer (1 μM), 0.7 µL; template cDNA, 1 µL; and RNase-free water added to reach the final volume. The reaction conditions were as follows: 95 °C 120 s (s), followed by 40 cycles of 5 s at 95 °C and 30 s at 60 °C. All experiments were repeated three times. The relative expressions of target genes were calculated using the formula 2^−ΔΔCT^ after normalisation to GAPDH. All experiments were repeated three times.

### 2.7. Statistical Analysis

All statistical analyses were performed using the SAS 9.0 system (SAS Institute Inc., Cary, NC, USA) for Windows. The Pearson correlation coefficient was used to assess the correlation between RNA-seq data and RT-qPCR data for the target genes.

## 3. Results

### 3.1. Growth Kinetics of FPLV in F81 Cells

**Figure 1:**
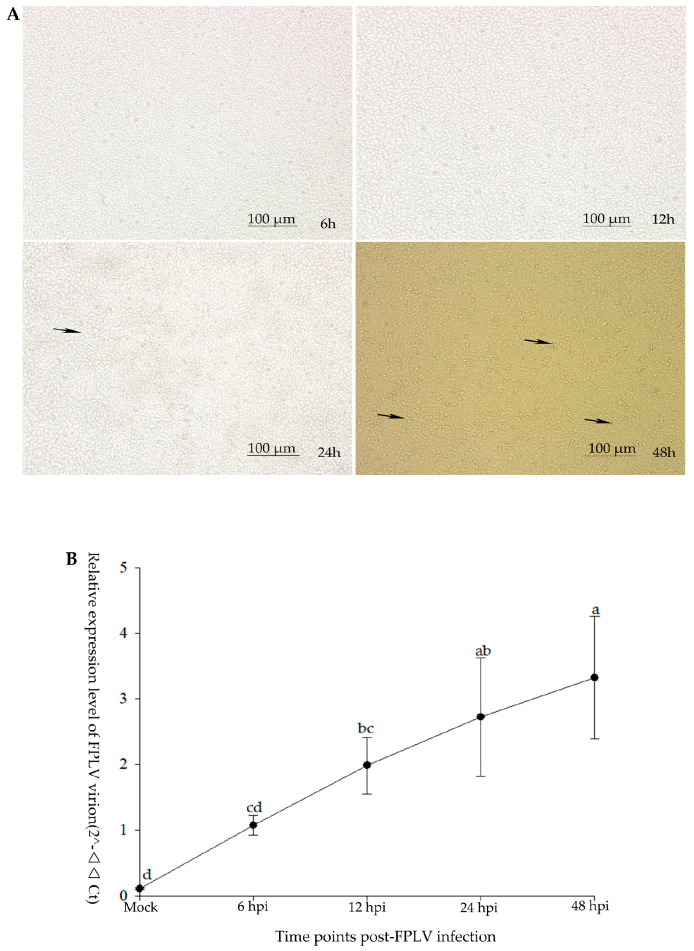
The growth kinetic analysis of FPLV infection in F81 cells. (**A**) Observation of cytopathic effect (CPE, indicated by black arrows) on FPLV-infected F81 cells in different hours post-infection (6 hpi, 12 hpi, 24 hpi, and 48 hpi). (**B**) Quantitative-PCR analysis of transcript levels of FPLV over time post-infection. Different lowercase letters indicate significant differences at the 0.05 level among different time points post-FPLV infection.

At the early period of FPLV infection (6–12 hpi), there were no macroscopic cytopathic effects (CPE) observed in FPLV-infected F81 cells. At 24 hpi, the infected F81 cells showed slight CPE. At 48 hpi, the CPE becomes macroscopic (Figure 1A). In addition, the transcript level of FPLV was detected using RT-qPCR with a pair of primers that target the VP2 sequences of FPLV (Table 2). The results showed that virus proliferation began as soon as 6 hpi and continued to increase until 48 hpi (Figure 1B).

### 3.2. Transcriptome Sequencing and Mapping with Reference Genome

Following sequencing, the raw data were filtered, and the percentage of clean reads was over 94% for all samples. Furthermore, the Q30 (%) was impressively high, reaching 94% for all samples, except for one instance with 93.55% (Table 3).

High-quality sequences (clean data) were subsequently mapped to the reference genome (Table 4). Approximately 91% of the clean reads were successfully mapped to the reference genome, with over 95% of these map events being uniquely mapped. Most of these uniquely mapped events occurred within genes (exceeding 95%), primarily in exons (over 91%), with only a small proportion mapping to intergenic regions. The distribution of reads across known gene types is presented in Table 5. Most reads primarily correspond to protein-coding sequences, followed by lncRNA, rRNA, and tRNA in decreasing order of prevalence.

### 3.3. Correlation Analysis of Gene Expression Levels Between Samples

**Figure 2:**
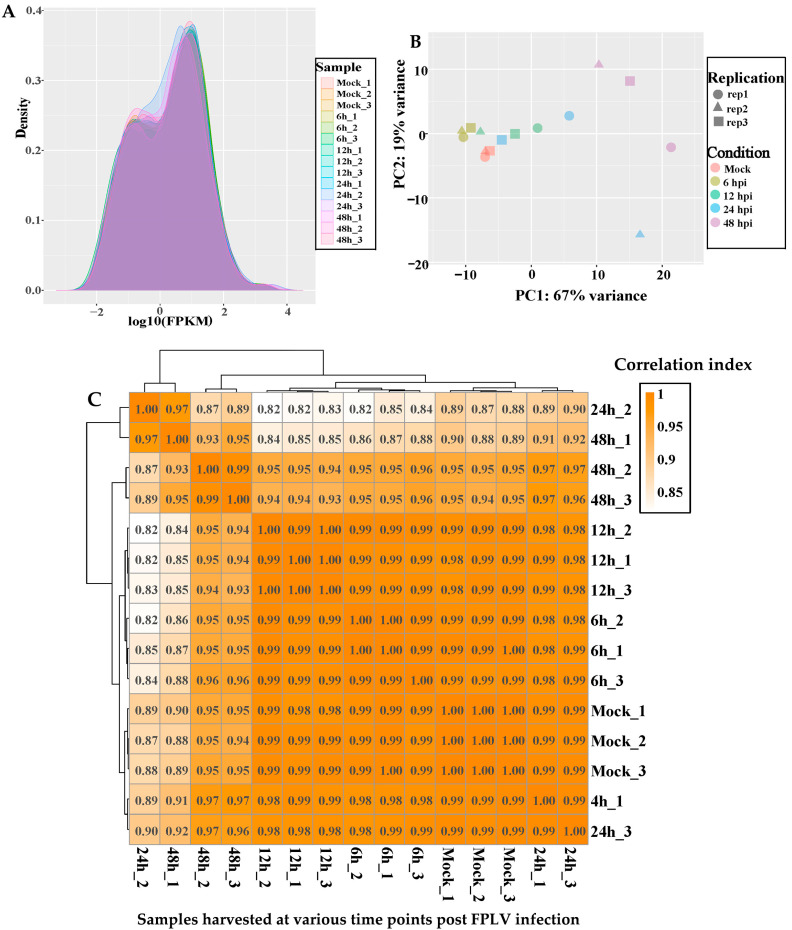
Analysis of gene expression for different FPLV infection times. (**A**) Fragments per kilobase per million fragments (FEAM) density distribution (density plot) of RNA-seq data. (**B**) Principal component analysis (PCA) of gene expression. (**C**) Inter-sample correlation analysis of gene expression.

To ensure the comparability of gene expression levels across different genes and samples, FPKM (fragments per kilobase per million fragments) was employed for standardising gene expression levels (normalisation). Generally, genes with an FPKM value greater than 1 are considered expressed (Figure 2A).

PCA is a dimensionality reduction technique used to select critical biological parameters. PCA can gather similar samples together, and the closer the distance is, the higher the similarity between samples. In this study, the expression patterns of gene expression between samples at 6 hpi (rep1, rep2, rep3), 12 hpi (rep1, rep2, rep3), or 24 hpi (rep1, rep3) were analysed, indicating higher similarity between these samples. In comparison, samples at 24 hpi (rep 2) and 48 hpi (rep 1, rep 2, rep 3) were distributed relatively scattered, indicating differences in gene expression between these samples (Figure 2B).

The correlation of gene expression levels between samples is a crucial indicator for assessing the reliability of experiments and the rationality of sample selection, which was represented using the Pearson correlation coefficient. If the correlation coefficient falls within the range of 0.80 to 1.00, the gene expression levels between samples are considered highly correlated. In this study, all Pearson correlation coefficients between the different samples were found to be between 0.82 and 1 (Figure 2C), indicating that the cleaned data were suitable for analysing differentially expressed genes (DEGs).

### 3.4. Analysis of Differentially Expressed Genes (DEGs) upon FPLV Infection

**Figure 3:**
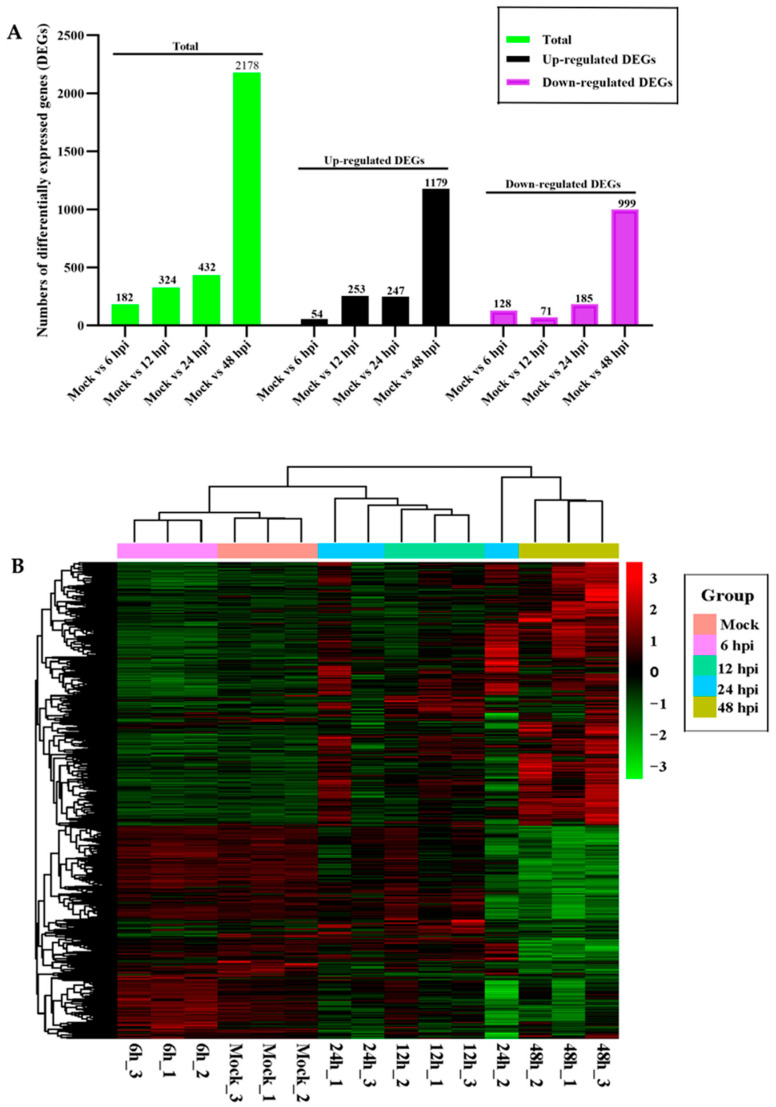
Analysis of differentially expressed genes (DEGs) upon FPLV infection. (**A**) Statistical analysis of differentially expressed genes (DEGs) (column plot). (**B**) Cluster analysis of differentially expressed genes (DEGs) (heat map).

The statistical results for differentially expressed genes (DEGs) between various treated groups are presented in Figure 3A. Based on a comparison to the control group (CK), a total of 3116 DEGs were identified in samples collected at various hours post-infection (hpi). This includes 182 DEGs (54 up-regulated and 128 down-regulated) at 6 hpi, 324 DEGs (253 up-regulated and 71 down-regulated) at 12 hpi, 432 DEGs (247 up-regulated and 185 down-regulated) at 24 hpi, and, significantly, 2178 DEGs (1179 up-regulated and 999 down-regulated) at 48 h post-infection.

Cluster analysis was used to determine the expression patterns of DEGs under different experimental conditions. Genes with high expression correlation across samples are grouped into the same category. The horizontal axis represents genes, with each column corresponding to one sample. Red indicates highly expressed genes, and green shows lowly expressed genes (Figure 3B).

### 3.5. Gene Ontology (GO) Enrichment Analysis of Differentially Expressed Genes (DEGs)

**Figure 4:**
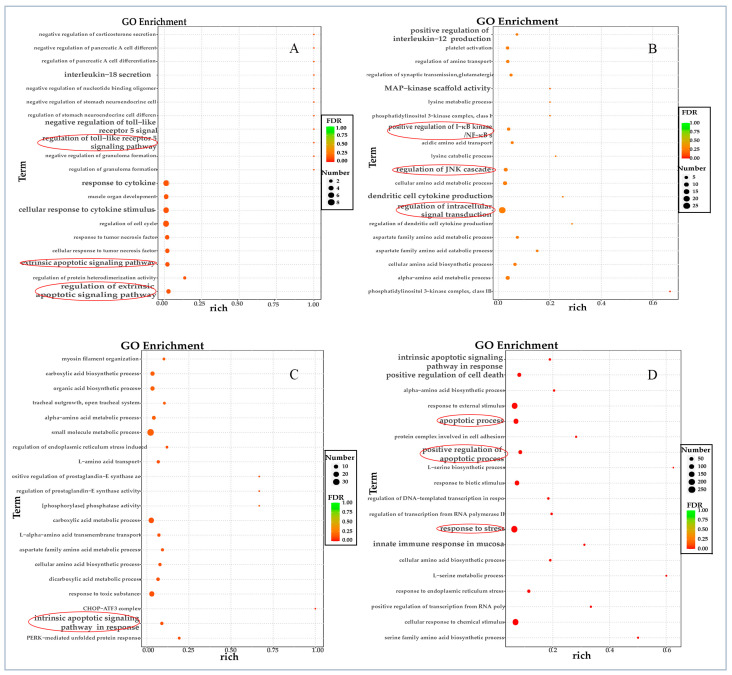
Gene ontology (GO) enrichment analysis of up-regulated genes at different time points post-FPLV infection (hpi). (**A**) GO analysis of up-regulated genes at 6 hpi. (**B**) GO analysis of up-regulated genes at 12 hpi. (**C**) GO analysis of up-regulated genes at 24 hpi. (**D**) GO analysis of up-regulated genes at 48 hpi.

In the early stages of viral infection (6 hpi), up-regulated genes are primarily enriched in regulating the cell cycle and the extrinsic apoptotic signaling pathway. A limited number of these genes were found to be involved in the Toll-like receptor 5 (TLR 5) signaling pathway and interleukin-18 secretion (Figure 4A). As the infection progressed to 12 hpi, a greater number of up-regulated genes were enriched in the regulation of intracellular signal transduction, particularly in the NF-κB and JNK signaling pathways. Additionally, an increase in the production of interleukin-12 and platelet activation is noted (Figure 4B).

After 24 h of infection, there is a shift in the function of up-regulated genes to small molecule metabolic processes or responses to toxic substrates (Figure 4C). As the disease progressed to 48 h, there is an enrichment of up-regulated genes involved in responses to external stimuli and stress, apoptosis processes, and positive regulation of cell death (Figure 4D). This progression suggests a complex interplay between the virus and the host’s cellular responses over time.

**Figure 5:**
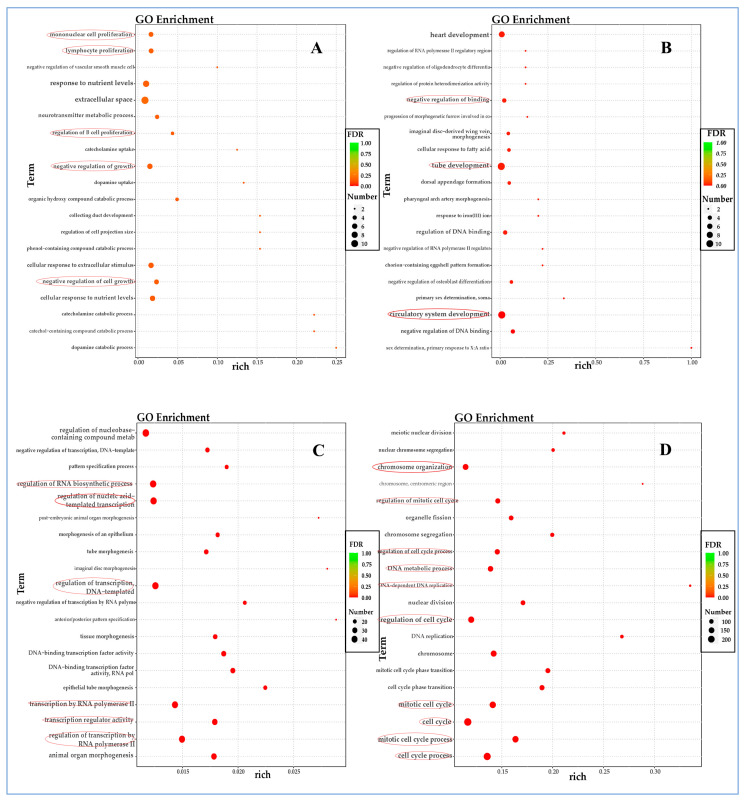
Gene ontology (GO) enrichment analysis of down-regulated genes at different time points post-FPLV infection (hpi). (**A**) GO analysis of down-regulated genes at 6 hpi. (**B**) GO analysis of down-regulated genes at 12 hpi. (**C**) GO analysis of down-regulated genes at 24 hpi. (**D**) GO analysis of down-regulated genes at 48 hpi.

The down-regulated differently expressed genes (DEGs) are predominantly enriched in the processes related to the immune response to virus invasion, such as mononuclear cell, lymphocyte, and B-cell proliferation, during the early stages of virus infection (6 hpi). As the infection progressed, the down-regulated DEGs began to be enriched in cellular functions, specifically negative regulation of binding and tube development at 12 hpi (Figure 5B). This trend continued in subsequent periods of virus infection, with an enrichment in transcription processes at 24 hpi (Figure 5C) and the cell cycle at 48 hpi (Figure 5D).

### 3.6. Kyoto Encyclopedia of Genes and Genomes (KEGG) Analysis of Differentially Expressed Genes (DEGs)

**Figure 6:**
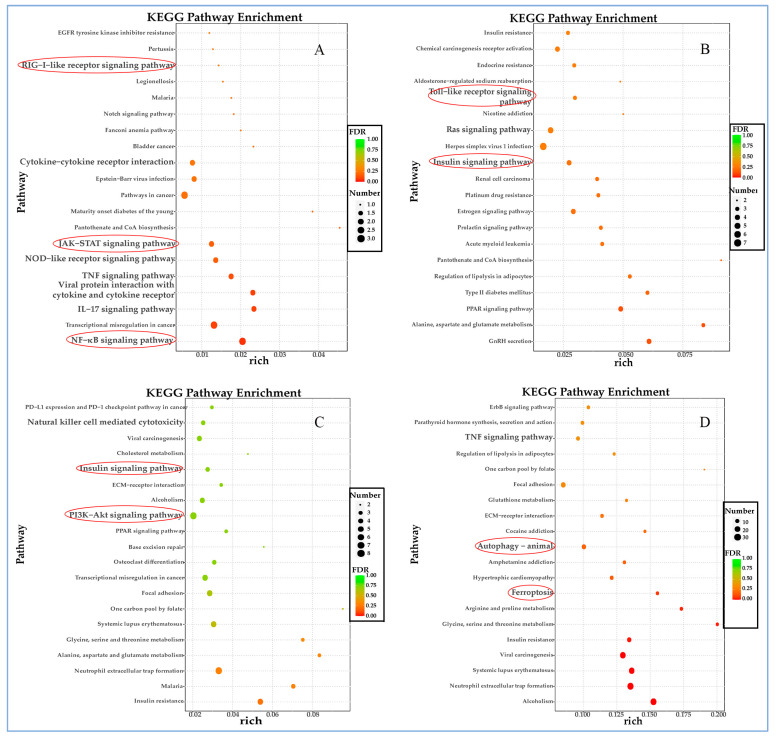
Kyoto Encyclopedia of Genes and Genomes (KEGG) analysis of up-regulated DEGs at different time points post-FPLV infection. (**A**) KEGG pathways of up-regulated DEGs at 6 hpi associated with immunity. (**B**) KEGG pathways of up-regulated DEGs at 12 hpi associated with immunity. (**C**) KEGG pathways of up-regulated DEGs at 24 hpi related to immunity. (**D**) KEGG pathways of up-regulated DEGs at 48 hpi related to immunity.

Multiple innate immune signaling pathways are activated after 6 h of infection of F81 cells with FPLV, such as NF-κB, NLR, RLR, TNF, IL-17, and JAK-STAT signaling pathways (Figure 6A). Genes associated with TLR, TNF, and Ras signaling pathways are up-regulated up to 12 h post-infection (hpi) (Figure 6B). Transcript abundance for genes involved in insulin; phosphatidylinositol 3-kinase (PI3K)-serine/threonine protein kinase (AKT) (PI3K-AKT); mitogen-activated protein kinases (MAPK) signaling pathway; and Th1, Th2, and Th17 cell differentiation increased at this time point (24 hpi). Additionally, genes associated with natural killer cell-mediated cytotoxicity and amino acid metabolism are also up-regulated (Figure 6C). At 48 h post-infection, the TNF and insulin signaling pathways remain continuously activated, and autophagy and ferroptosis, a novel cell death mode, are observed (Figure 6D).

**Figure 7:**
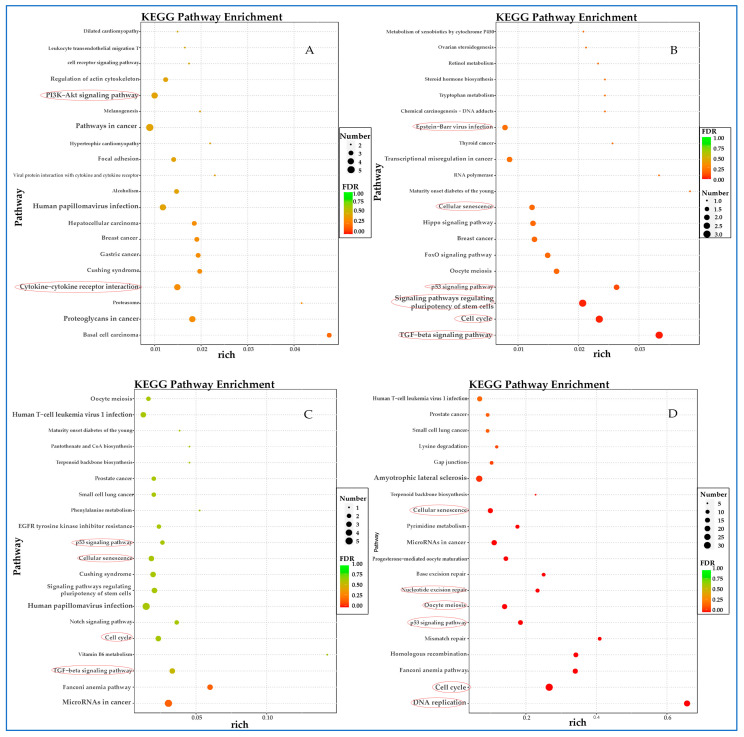
Kyoto Encyclopedia of Genes and Genomes (KEGG) analysis of down-regulated DEGs at different time points post-FPLV infection (hpi). (**A**) KEGG pathways of down-regulated DEGs at 6 hpi. (**B**) KEGG pathways of down-regulated DEGs at 12 hpi. (**C**) KEGG pathways of down-regulated DEGs at 24 hpi. (**D**) KEGG pathways of down-regulated DEGs at 48 hpi.

The results of the KEGG analysis for the down-regulated DEGs are presented in Figure 7. During the early stage (6 hpi) of virus–host interaction, the down-regulated DEGs were primarily enriched in the PI3K-Akt signaling pathway and cytokine–cytokine receptor interaction (Figure 7A). As the experiment progressed, the down-regulated DEGs played a significant role in cellular senescence, the p53 signaling pathway, and the cell cycle at 12 (Figure 7B) and 24 hpi (Figure 7C). This trend persisted throughout the duration of the experiment (48 hpi) (Figure 7D).

### 3.7. Validation of the RNA-Seq Results

**Figure 8:**
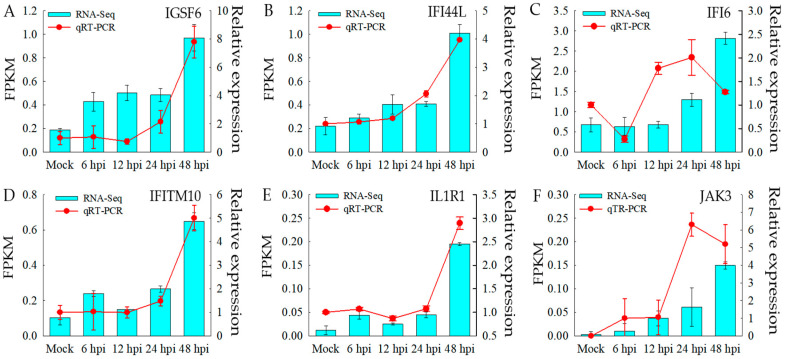
Verification of expression of different genes based on real-time fluorescence quantitative PCR. (**A**) Validation of up-regulated DEGs (IGSF6). (**B**) Validation of up-regulated DEGs (IF144L). (**C**) Validation of up-regulated DEGs (IFI6). (**D**) Validation of up-regulated DEGs (IFITM10). (**E**) Validation of up-regulated DEGs (IL1R1). (**F**) Validation of up-regulated DEGs (JAK3).

The expression of six up-regulated differentially expressed genes (DEGs) related to the innate immune response pathways was validated using real-time RT-PCR (Figure 8). For example, IFI44L gene expression was up-regulated by 1.31, 1.87, 2.17, and 4.92 times at 6 h, 12 h, 24 h, and 48 h compared to the baseline in the RNA-seq data, respectively. At the same time, the corresponding values in the RT-qPCR results are 1.07, 1.19, 2.05, and 3.95 times, respectively. Moreover, the expression patterns of IGSF6, IFITM10, IL1R1, JAK3, and IFI6 are in line with the trends observed in the transcriptome sequencing data.

The significant differences analysis of target genes by RT-qPCR validation at different time points were shown in Table 6. Most of target genes showed significant difference at 24 and 48 hpi (*p* < 0.05)

## 4. Discussion

Feline panleukopenia virus (FPLV) was discovered over 100 years ago, yet nearly all studies on FPLV have primarily concentrated on the virus’s activity [17], genetic characteristics [18,19,20], or vaccine development [21,22], with relatively few reports examining host–pathogen interactions between FPLV and host cells.

In this study, we perform an RNA-seq analysis on FPLV-infected feline kidney cells (F81) to illustrate host–virus interactions between FPLV and F81 cells during FPLV infection, based on a Chinese FPLV variant carrying a VP2 A91S substitution. Overall, 3116 genes were identified to have experienced significant changes, with 1733 genes being up-regulated and 1383 genes being down-regulated simultaneously. Further analysis indicated that the down-regulated DEGs were predominantly enriched in negative regulation of the cell cycle, cell growth, or cell senescence (Figure 5 and Figure 7), in contrast to the up-regulated DEGs, which were more enriched in processes related to innate immunity (Figure 4 and Figure 6). The primary focus of this study was on innate immunity of FPLV; therefore, we paid more attention on the up-regulated differentially expressed genes (DEGs) associated with immune-related pathways.

Viral infections trigger an immune response in host cells, leading to the release of cytokines, such as IL-17, TNF, and various T helper cell types (Th1, Th2, and Th17), at 6 and 12 hpi. The IL-17 and TNF signaling pathways play critical roles in protecting host cells from viral attacks. They work together to combat feline panleukopenia virus by enhancing the host’s immune response, potentially influencing immune and inflammatory responses, regulating cell death, and modulating the activity of immune cells.

Meanwhile, the extrinsic apoptotic, NF-κB, JAK-STAT, and Toll-like receptor (TLR) signaling pathways were activated based on several genes associated with these pathways, including CLU, TRAF1, TRIM13, IL32, JAK3, TLR2, TLR3, TLR7, and TLR10, despite the absence of macroscopic cytopathic effects (CPE) on F81 cells (6–12 hpi). Compared to other feline viruses, such as FHV-1, TLR2 and TLR4 were also up-regulated at early stages (3 hpi vs. 6 hpi) of virus infection [11]. TLRs play a crucial role in antiviral immunity by serving as the first line of defence against viral invasion [23]. In this study, the up-regulation of TLR3 and TLR7 may initiate the activation of MyD88 and TRIF, leading to an overproduction of IFN to defend against FPLV and counteract viral absorption.

As the infection progressed, the CPE of FPLV on F81 cells gradually became more evident from 24 to 48 hpi, accompanied by the activation of the intrinsic apoptotic signaling pathway and the positive regulation of the apoptotic process. This was indicated by the DEGs enriched in the autophagy (IL1R1, TNFSF10, TRIB3, PIK3R5, and IL32) and ferroptosis (BRIC7, ACSL1, ACSL3, ACSL4, and ACSL5) pathways that were up-regulated. Autophagy is a degradation process that maintains cellular homeostasis, ensuring cellular survival under various stress conditions, such as pathogenic infections. As reported, autophagy can promote parvovirus replication and induce non-apoptotic cell death [24,25].

In addition, this study found that IFI6, IFNB1, IGSF6, ISG15, MAPK10, IL1R1, IRF7, IFI44L, and IFTIM10 were also up-regulated and associated with innate immunity. However, there are no reports delineating the functional roles of these genes in host–pathogen interactions with FPLV, although some researchers have reported they play crucial roles in innate immunity against other viruses, such as ISG15 [26,27], IFI44L [28], IFTIM [29], and IFI6 [30]. Therefore, it is essential to conduct in-depth research on the role of these genes in the host infection of FPLV and anti-infection processes in the host.

Our RNA-seq analysis provided valuable insights into differential gene expression patterns associated with FPLV infection. A significant limitation of this study is the lack of functional follow-up experiments to confirm transcriptomic predictions. Such assays, such as measuring cytokine production or assessing signaling pathway activation, are essential for establishing the biological significance of the identified differentially expressed genes (DEGs). Therefore, further research is needed to deepen our understanding of FPLV–host interactions and better evaluate how FPLV infection impacts FPLV–host interactions, as well as assess how FPLV infection affects host gene expression and immune responses.

Overall, the results of this in vitro study have revealed a distinct transcriptomic signature characteristic of FPLV infection, with the identification of up-regulated genes associated explicitly with the antiviral defence response as well as other broadly expressed genes. The findings demonstrate that FPLV infection triggers a multifaceted and complex biological response in F81 cells, with a central focus on antiviral immunity. This study highlights the critical role of these processes in mediating viral clearance and underscores their importance in understanding the host’s antiviral defence mechanisms.

## 5. Conclusions

In summary, we present the first reported study on the systematic transcriptome analysis of feline kidney cells (F81) during the initial phases of feline panleukopenia virus (FPLV) infection using RNA-seq technology. Our findings reveal alterations in the host transcriptome during FPLV infection, suggesting that the virus activates immune responses in F81 cells and disrupts early defence mechanisms. These insights enhance our comprehension of FPLV–host interactions and pave the way for developing effective strategies to prevent and treat FPLV infections.

## Figures and Tables

**Table 1:** The Felis catus reference genome.

Genome	GCF_018350175.1_F. catus_Fca126 _mat1.0_genomic.fna
Gene built by	https://www.ncbi.nlm.nih.gov/assembly/GCF_018350175.1 (accessed on 15 April 2023)
Base Pairs	2425747038

**Table 2:** RT-qPCR primer information.

Gene	Primer	Sequence (5′–3′)	Product Size (bp)
IGSF6	F	TCTCTTTTATGTTGGTGCTGCT	147
	R	CACAGGCTTCTTGGTTGCTC	
IFI44L	F	GCATCAGAGTTGGAGCTGGA	120
	R	CAGCCTTCCTCCCTGTTTCA	
IFI6	F	GACCTACATGGCTGTCGGAG	186
	R	CCACCACTAGCCCCGAG	
IFITM10	F	GGCCTACTCCCTCAAAGTTCG	124
	R	GATGATACAAGAGGCCGCCA	
IL1R1	F	TGGAGGATTATTTGCCAGTGGA	130
	R	TCTGTATTCTTGGCTACACAGGT	
JAK3	F	GCCCCCAGACCCAAAGAAAA	132
	R	GTCAGCGGGGATCTTGTGAA	
GAPDH	F	ACCATCTTCCAGGAGCGAGAT	141
	R	GATGATGACCCTCTTGGCCC	
FPLV	F	GAAGCGTCTACACAAGGGC	155

**Table 3:** RNA-seq data.

Sample	Reads No.	Clean Reads No.	Clean Reads (%)	Q20 (%)	Q30 (%)
Mock_1	45844098	43396820	94.66	98.09	94.64
Mock_2	46432876	43999136	94.75	98.08	94.46
Mock_3	48594575	45110578	94.59	98.01	94.35
6hpi_1	47041828	44555458	94.71	98.05	94.4
6hpi_2	44583130	42199820	94.55	98.08	94.53
6hpi_3	54755355	51851725	94.59	98.12	94.5
12hpi_1	52183988	49454432	94.78	98.06	94.37
12hpi_2	53055038	50253352	94.72	98.13	94.53
12hpi_3	41943112	39753022	94.8	97.91	94.02
24hpi_1	50211258	47555192	94.73	98.11	94.47
24hpi_2	45590044	43103750	94.54	97.77	93.9
24hpi_3	50799730	48133754	94.75	97.8	93.55
48hpi_1	49314514	45592438	94.47	98.2	94.95
48hpi_2	45585320	43244954	94.55	98.12	94.47
48hpi_3	45205355	43723212	94.52	98.02	94.35

**Table 4:** RNA-seq data mapping to the Felis catus reference genome.

Sample	Total Mapped/%	Mapped Events		
		Uniquely Mapped/%	Mapped Gene/%	Mapped Exon/%
Mock_1	95.65%	96.63%	96.21%	93.86%
Mock_2	95.86%	96.64%	96.39%	94.54%
Mock_3	95.50%	96.65%	96.22%	93.59%
6hpi_1	95.99%	96.64%	96.47%	93.92%
6hpi_2	96.03%	96.60%	96.51%	93.83%
6hpi_3	96.01%	96.46%	96.62%	94.71%
12hpi_1	95.82%	96.43%	96.01%	93.68%
12hpi_2	96.52%	96.52%	96.38%	93.35%
12hpi_3	95.23%	96.50%	96.09%	93.31%
24hpi_1	95.34%	96.56%	95.80%	93.13%
24hpi_2	95.84%	95.84%	96.47%	95.65%
24hpi_3	96.37%	96.37%	96.96%	96.35%
48hpi_1	96.16%	96.16%	96.38%	95.52%
48hpi_2	95.74%	95.74%	96.04%	92.51%
48hpi_3	96.45%	96.45%	95.49%	91.55%

**Table 5:** The distribution of mapped reads according to known types of genes.

Sample	Guide_RNA	snoRNA	Protein Coding	tRNA	snRNA	rRNA	MiscRNA	lncRNA
Mock_1	54	3230	16616396	1270	265	7297	1045	143635
Mock_2	45	2596	17062110	1557	282	8387	1092	138324
Mock_3	77	3033	17638131	1111	294	7774	1054	158523
6hpi_1	79	2918	17274359	1113	283	8083	1097	137411
6hpi_2	51	2833	16391432	1150	233	7256	1044	131000
6hpi_3	54	3020	20318234	1256	272	7349	1235	154339
12hpi_1	77	2190	18856856	2373	326	18854	1165	171334
12hpi_2	67	2871	19143685	1972	350	13840	1023	157359
12hpi_3	67	2095	15052592	1664	289	12232	1019	135855
24hpi_1	77	2342	17556534	2462	342	29830	1090	178996
24hpi_2	38	1437	16442456	1501	232	27734	686	166990
24hpi_3	41	2162	18304926	1666	166	18533	925	124312
48hpi_1	80	1872	17420227	1653	213	67920	729	174961
48hpi_2	40	2056	14586176	2333	240	127689	723	137485
48hpi_3	55	2593	15556719	1947	358	46398	878	169124

**Table 6:** Significant differences analysis of target genes by RT-qPCR validation at different time points.

Time	IGSF6	IFI44L	IFI6	IFITM10	IL1R1	JAK3
Mock	1.000 c	1.000 c	1.000 ab	1.000 b	1.000 b	1.000 c
6hpi	1.070 c	1.065 c	0.273 b	1.028 b	1.071 b	0.789 d
12hpi	0.748 c	1.192 c	1.777 ab	0.996 b	0.870 b	1.254 bc
24hpi	2.148 b	2.060 b	2.009 a	1.478 b	1.077 b	1.434 b
48hpi	7.794 a	3.966 a	1.273 a	5.003 a	2.891 a	2.057 a

Note: Different lowercase letters indicate significant differences at the 0.05 level.

## Data Availability

All data generated or analyzed during this study are included in this published article.
